# Serological evaluation of Crimean-Congo Hemorrhagic fever in humans with High-Risk professional exposure and in residual sera collected in 2022-2023 across Corsica (France)

**DOI:** 10.1016/j.onehlt.2025.101020

**Published:** 2025-03-25

**Authors:** Paloma Kiwan, Morena Gasparine, Dorine Decarreaux, Lisandru Capai, Shirley Masse, Miša Korva, Tatjana Avšič-Županc, Jean Canarelli, Marie-Helene Simeoni, Xavier de Lamballerie, Remi Charrel, Alessandra Falchi

**Affiliations:** a Unité des Virus Émergents (Aix Marseille Université, Università di Corsica, IRD 190, INSERM 1207, IRBA), Marseille, France; b Institute of Microbiology and Immunology, Faculty of Medicine, University of Ljubljana, Ljubljana, Slovenia; c Laboratoire de Biologie Médicale CCF, Ajaccio, France; d Laboratoire de Biologie Médicale 2A2B, Corte, France; e Centre National de Référence des Arbovirus, Marseille, France

**Keywords:** Crimean-Congo Hemorrhagic Fever Virus (CCHFV), Tick-borne virus, Seroprevalence, Tick bite, High-risk, Human populations, Corsica

## Abstract

**Background:**

The Crimean-Congo hemorrhagic fever virus (CCHFV) is a tick-borne pathogen known to cause severe viral hemorrhagic fever.

**Aim:**

We aimed to evaluate the potential circulation of CCHFV in Corsica through a serosurvey, including anonymized residual sera (RS) and high-risk individuals exposed to animals and/or tick bites due to occupational activities.

**Methods:**

This cross-sectional study involved two groups: anonymized RS from medical biology laboratories and sera from high-risk individuals (slaughterhouse workers, veterinary professionals, animal farmers, and rangers) collected across Corsica during 2022–2023. Antibodies targeting the CCHFV viral nucleoprotein were detected using a double-antigen ELISA. ELISA-positive samples underwent neutralizing antibody testing. Sociodemographic and epidemiological data were collected using a structured questionnaire in the high-risk group.

**Results:**

Total anti-CCHFV seropositivity was of 0.08 % (*n* = 2) [95 % Confidence Interval (CI): 0.06–0.09] in RS and of 0.50 % (*n* = 1) [95 % CI: 0.43–0.56] in high-risk groups (*p* < 0.01). Lifetime tick-bites was reported by 65.9 % (*n* = 118) of respondents, with higher rates among farmers (Odds Ratio (OR) = 3.4; 95 % CI 1.4–8.5) and participants with >10 years of occupational exposure (OR = 3.8; 95 % CI 1.7–8.5).

**Conclusions:**

This study provides initial evidence of human exposure to CCHFV in Corsica, with rates consistent with those observed in other Western European regions. Our results indicate a risk of CCHF among the Corsican population, particularly among farmers and slaughterhouse workers. Continuous surveillance and public education are essential to mitigate this risk, especially among these targeted groups and healthcare professionals, ensuring prompt diagnosis and prevention of potential outbreaks.

## Graphical abstract

**Figure:**
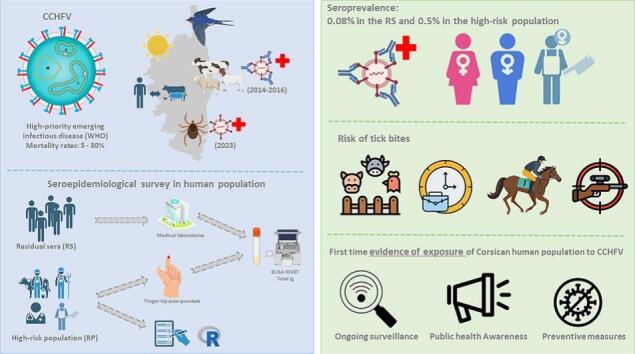

## 1 Introduction

Crimean-Congo hemorrhagic fever virus (CCHFV) is a tick-borne Orthonairovirus with potential to cause severe disease in humans [1]. The wide distribution of its main vector, the *Hyalomma* ticks, and recent advances in molecular virus detection, has led to extensive mapping of the viral presence [2]. Since 2014, the World Health Organization classified CCHF as a high-priority emerging infectious disease due to the absence of licensed vaccines, approved specific medical treatments and a case fatality rate (CFR) of up to 30 %, depending on healthcare infrastructures and early hospital referral [3]. Humans are considered incidental hosts [1], becoming infected through tick bites, contact with blood and/or body fluids or tissues of a viremic animals or humans, or through handling and slaughtering infected animals [4]. CCHFV poses an occupational risk to individuals working in land and animal management, such as farmers, slaughterhouse workers, rangers, hunters, and veterinarians, as well as to those exposed to human contaminated body fluids such as healthcare workers [4].

In Western Europe, a total of 18 human cases of CCHF have been reported in the Iberian Peninsula since 2013, with 17 cases in Spain and one recently in Portugal [5,6]. Although no human cases have been diagnosed in France to date, there is a risk in southern regions, particularly in the southwestand Corsica, a French Mediterranean island. Corsica has been identified as a risk area for CCHF emergence due to: i) serological evidence of CCHFV exposure in domestic livestock [7], ii) presence of the main vector, *Hyalomma marginatum,* which is now abundant and widespread [8], and iii) the recent detection and genome sequencing confirmation of CCHFV RNA (Africa 1 genotype) in ticks collected from cattle [9]. To date, no human CCHF case has been reported in the whole of France. However, symptomatic cases represent only a small proportion of the total infected population, as about 90 % of CCHFV infections in humans are asymptomatic or cause non-specific clinical signs [10]. Thus, the cross-sectional study presented here was carried out to estimate human population exposure to CCHFV using sera obtained from i) medical biology laboratories and ii) high-risk occupational populations across Corsica during 2022–2023. We also reported on tick-bite exposure in high-risk groups.

## 2 Materials and methods

### 2.1 Study design

This cross-sectional study involved two groups of samples. The first group consisted of anonymized residual sera (RS) collected between October 2022 and July 2023. RS refers to serum samples obtained from individuals for routine screening or clinical management by 11 medical laboratories. To ensure representation of age groups, RS samples were selected, targeting an appropriate number from each 10-year age group, with no samples collected from minors (ranging from 18 to 19 years to ≥90 years). This sampling strategy aimed to reflect the actual age distribution of the Corsican population [11]. Detailed demographic information, including age, sex, and sampling location (identified by postal code), were recorded for each participant, to ensure confidentiality and anonymity. All duplicates were excluded from the database based on the criteria of date of birth, gender and laboratory.

The second group consisted of individuals at high risk of exposure to animals and/or tick bites due to their occupational activities: slaughterhouse workers, veterinarians, animal farmers and rangers. Sera were collected from October 2022 to September 2023. Participants provided informed consent authorizing anonymous collection of both capillary blood sample using a fingerstick procedure (self-sampling), which were then centrifuged to obtain serum, followed by questionnaire administration. People living in Corsica for less than one year were excluded. The questionnaire covered sociodemographic factors, occupational activities, outdoor activities, previous exposure to tick bites in the work environment and during leisure time, perception of tick-borne diseases (TBDs), and implemented preventive measures.

All sera samples were subsequently stored at −20 °C for further analyses.

### 2.2 Serological assay

#### 2.2.1 Elisa serological screening

All sera were screened for the presence of total antobodies against the CCHFV nucleocapsid protein using the ID Screen CCHF Double Antigen Multi-species ELISA (IDvet, Grabels, France) following the manufacturer's instructions. The microplate's optical density (OD) was measured at 450 nm using a plate reader (Thermo Scientific™ Multiskan™, Waltham, MA, USA). Positive samples were subjected to a second round of testing to verify the consistency of the findings.

#### 2.2.2 Neutralization assay

ELISA positive samples were tested for the presence of neutralizing antibodies. Briefly, Vero E6 cells (ATTC CRL-1586) were seeded at 10^5^ cells/well in 96-well plates (TPP, 92196) supplemented with growth medium DMEM with GlutaMAX supplement (Thermo Fisher Scientific, 61,965,026) and 10 % FBS (Euroclone, ECS0180L) and incubated for 24 h at 37 °C in 5 % CO_2_. Two-fold serial dilutions (initial dilution was 1:10) of heat-inactivated sera samples (56 °C, 30 min) were incubated with 10^4^ TCID_50_ of CCHFV (CCHFV strain Kosovo Hoti; deposited in the EVA-GLOBAL European Virus Archive under reference number Ref-SKU: 007 V-02504) for 1 h at 37 °C. Then, 50 μL of the serum dilution-virus mixture was inoculated in triplicate into a 96-well plate containing an 80 % confluent Vero E6 monolayer. The plates were incubated for 5 days at 37 °C before being fixed with 100 μL of 4 % formaldehyde. Afterwards, plates were examined microscopically for cytopathic effect (CPE) and the cell monolayer was additionally visualized by crystal violet staining. The neutralization endpoint titer was defined as the endpoint serum dilution that inhibited CCHFV-induced CPE in at least two out of three parallels. Positive and negative control sera were included in each plate.

#### 2.2.3 Ethical consideration

For the RS sample, the study adhered to reference methodology MR-004, under French law 2016–41 of January 26, 2016, pertaining to the modernization of the French health system. Authorization for the study was obtained on April 20, 2020, and registration with the French Data Protection Authority (Commission Nationale de l'Informatique et des Libertés (CNIL)) was completed under reference CNIL: 2217594 v 0. Regarding the high-risk population, the study was classified as research involving human subjects of category 2, under Article L. 1121–1 of the French Public Health Code (reference 2022–63) and was declared compliant with reference methodology MR-001(CNIL: 2226544). Written informed consent was obtained from participants after providing detailed information about the study's purpose and procedures.

### 2.3 Statistical analysis

No data were available regarding CCHFV seroprevalence in the French human population, which prompted us to estimate based on rates reported in Spain (0.58–1.16 %) and Portugal (1.1 %) [12,13]. For the RS group, a minimum of 457 sera was calculated assuming the following: an a priori 0.5 % seroprevalence mean value of anti-CCHFV antibodies, a confidence level of 95 %, and a maximum allowable error in the prevalence of 3 %. For high-risk groups, a minimum of 139 sera was calculated assuming a priori 1 % seroprevalence of anti-CCHFV antibodies (due to hypothetical higher exposure to animals and/or tick bites with respect to the RS sample), a confidence level of 95 %, and a maximum allowable error in the prevalence of 5 %.

Descriptive statistics included counts and percentages for categorical variables, as well as medians and ranges for continuous variables. Associations between categorical variables were tested using the χ2 test and the Fisher's exact test. The Wilcoxon rank-sum test was used to compare age distributions between individuals by “tick bite” declaration (Yes vs. No) within the high-risk groups. A *Z*-test was used to compare seropositivity between RS and high-risk groups. Statistical significance was defined as a two-tailed *p*-value < 0.05.

Two outcomes were estimated: i) CCHFV seroprevalence in RS and high-risk group samples, and ii) self-reported tick bite rates in high-risk groups. The first outcome measure was the prevalence of CCHFV infection, defined as the presence of CCHFV antibodies detected at least by the ELISA assay. Seroprevalence was estimated with a 95 % confidence interval (CI). The second outcome was the proportion of participants who experienced tick bites in high-risk groups. This was computed as the proportion of participants who had experienced tick bites in their lifetime and during the past 12 months.

As slaughterhouse workers are not at risk of tick bites in their working environment, self-reported occupational tick bites exposure was investigated for farmers, rangers and veterinarians. Meanwhile, tick bite exposure related to non-occupational activities was assessed for all four high-risk populations. Factors associated with self-reported tick-bites were analyzed using a binary logistic regression model. All the following variables with *p*-values <0.2 in the univariable analysis were considered in the multivariable analysis: occupational activity, career duration, hiking, equitation, fishing, hunting, evisceration after hunting activities and awareness of tick-borne human and animal diseases. A step-by-step backward elimination procedure identified independent covariates associated with self-reported tick bites in the multivariable analysis (*p*-value <0.05). Odds ratios (ORs) and 95 % CI were estimated for each variable analyzed. The data analyses were conducted using RStudio version 4.2.2 and R packages (The R Foundation for Statistical Computing, Vienna, Austria) [14].

## 3 Results

A total of 2514 RS were collected. The median age was 52 years (Min = 15; Max = 104), and 60.9 % (*n* = 1530) were women (Additional file). Data regarding socio-demographic and occupational characteristics of the high-risk populations subdivided according to job, are reported in Table 1 and Table 2. A total of 201 participants (6.5 % (*n* = 13) slaughterhouse workers, 20.9 % (*n* = 42) veterinary professionals, 36.3 % (*n* = 73) rangers, 36.3 % (n = 73) farmers, of which 65.8 % (*n* = 48) were involved in cattle and equine farming) who completed a questionnaire and underwent interpretable serological analysis were included in this study. The median age of the 201 participants was 39 years (Min = 18; Max = 85), and 54.7 % (*n* = 110) were men (Table1).

**Table 1:** Description of participant characteristics in the high-risk populations.

Characteristic	Farmer, N = 73^1^	Slaughterhouse worker, N = 13^1^	Rangers, N = 73^1^	Veterinary professionals, N = 42^1^	Overall, N = 201^1^
**Age** (years)	42 [18–85]	32 [26–53]	40 [20–66]	38 [23–67]	39 [18–85]
**Age group**					
18–39	33 (45.2 %)	9 (69.2 %)	36 (49.3 %)	24 (57.1 %)	102 (50.7 %)
40–49	18 (24.7 %)	2 (15.4 %)	20 (27.4 %)	11 (26.2 %)	51 (25.4 %)
50–69	19 (26.0 %)	2 (15.4 %)	17 (23.3 %)	7 (16.7 %)	45 (22.4 %)
70–89	3 (4.1 %)	0 (0.0 %)	0 (0.0 %)	0 (0.0 %)	3 (1.5 %)
**Sex**					
Man	34 (46.6 %)	13 (100.0 %)	52 (71.2 %)	11 (26.2 %)	110 (54.7 %)
**Education**					
No diploma	5 (6.9 %)	8 (61.5 %)	1 (1.4 %)	0 (0.0 %)	14 (7.0 %)
Primary school-secondary school	37 (50.7 %)	2 (15.4 %)	27 (37.0 %)	0 (0.0 %)	66 (32.8 %)
Higher education diploma	31 (42.51.7 %)	3 (23.1 %)	45 (61.6 %)	42 (100.0 %)	121 (60.0 %)
**Workplace district**					
Haute-Corse	58 (80 %)	5 (38.5 %)	51 (70 %)	20 (46.2 %)	134 (66.7 %)
Corse du Sud	15 (20 %)	8 (61.5 %)	22 (30 %)	22 (53.8 %)	67 (33.3 %)
**Career duration**					
<5 years	14 (19.2 %)	8 (61.5 %)	23 (31.5 %)	17 (40.5 %)	62 (30.8 %)
5–10 years	12 (16.4 %)	1 (7.7 %)	13 (17.8 %)	9 (21.4 %)	35 (17.4 %)
>10 years	47 (64.4 %)	4 (30.8 %)	37 (50.7 %)	16 (38.1 %)	104 (51.8 %)
**Hobbies**					
***Hiking***	48 (65.8 %)	2 (15.4 %)	57 (78.1 %)	36 (85.7 %)	143 (71.1 %)
***Equitation***	37 (50.7 %)	2 (15.4 %)	16 (21.9 %)	11 (26.2 %)	66 (32.8 %)
***Hunting***	24 (32.9 %)	1 (7.7 %)	24 (32.9 %)	3 (7.1 %)	52 (25.9 %)
**If hunting = yes, evisceration practices**	17 (70.8 %)	1 (100.0 %)	16 (66.7 %)	3 (100.0 %)	37 (71.2 %)
**If evisceration = yes, protection measures**	9 (52.9 %)	0 (0.0 %)	7 (43.8 %)	1 (33.3 %)	17 (45.9 %)
**Lack of tick precaution measures = yes**	63 (86.3 %)	13 (100.0 %)	55 (75.3 %)	41 (97.6 %)	172 (85.6 %)

1 Median [range]; n (%).

**Table 2:** Description of tick-bite history in the high-risk populations.

**Characteristic**	**Farmer**, N = 73^1^	**Slaughterhouse worker**, N = 13^1^	**Rangers**, N = 73^1^	**Veterinary professionals**, N = 42^1^	**Overall**, N = 201^1^
**Tick-bite history over a lifetime =** **yes**	53 (77.9 %)	8 (61.5 %)	40 (65.6 %)	17 (45.9 %)	118 (65.9 %)
Don't know	5	0	12	5	22
**For those who were bitten by ticks**	***N* = 53**	**N =** **8**	***N* =** **40**	**N =** **17**	**N = 118**
**Tick-bite frequency over lifetime**					
< 5 times	32 (60.4 %)	5 (62.5 %)	30 (75.0 %)	15 (88.2 %)	82 (69.5 %)
5–10 times	9 (17.0 %)	1 (12.5 %)	8 (20.0 %)	2 (11.8 %)	20 (16.9 %)
> 10 times	12 (22.6 %)	2 (25.0 %)	2 (5.0 %)	0 (0.0 %)	16 (13.6 %)
**Tick-bite symptoms**	10 (18.8 %)	2 (25.0 %)	5 (12.5 %)	5 (29.4 %)	22 (18.6 %)
**Tick removal methods**⁎					
Fingers	33 (62.3 %)	6 (75.0 %)	15 (37.5 %)	–	54 (45.8 %)
Tick removal tool	15 (28.3 %)	1 (12.5 %)	25 (62.5 %)	–	41(40.6 %)
Other	10 (18.9 %)	1 (12.5 %)	8 (20.0 %)	–	19 (18.8 %)
**Tick-bite last 12 months**	19 (35.8 %)	0 (0.0 %)	13 (32.5 %)	3 (17.6 %)	35 (29.7 %)
**Tick-bite frequency last 12 months**	**N** **=** **19**	**N** **=** **0**	**N** **=** **13**	**N** **=** **3**	**N** **=** **35**
< 5 times	17 (89.5 %)	0 (0.0 %)	13 (100.0 %)	3 (100.0 %)	33 (94.3 %)
5–10 times	1 (5.3 %)	0 (0.0 %)	0 (0.0 %)	0 (0.0 %)	1 (2.9 %)
> 10 times	1 (5.3 %)	0 (0.0 %)	0 (0.0 %)	0 (0.0 %)	1 (2.9 %)

1 n (%).

⁎ : percentages may not total 100 % due to participants selecting multiple responses.

### 3.1 Self-reported tick-bite exposure and risk factors among high-risk groups

As shown in Table 2, among the 201 participants, 89.1 % (*n* = 179) responded by “Yes” or “No” to the question: “*Have you been bitten by a tick at least once in your life in Corsica? (As you have noticed tick's attachment to your skin)”.* Twenty-two participants (10.9 %) did not know. Self-reported tick-bite history at least once in life was reported by 65.9 % (*n* = 118) of the 179 respondents, with 77.9 % (*n* = 53) being farmers, 65.6 % (*n* = 40) rangers, 61.5 % (*n* = 8) slaughterhouse workers, and 45.9 % (n = 17) veterinarians (*p* = 0.011). Tick-bite frequency was reported as “less than 5 times” for 69.5 % (*n* = 82) of respondents. Self-reported history of tick bites during the last 12 months was reported by 29.7 % (*n* = 35) of respondents, with 35.8 % (*n* = 19 of 53) of farmers; 32.5 % (*n* = 13 of 40) of rangers, 0.0 % (*n* = 0 of 8) of slaughterhouse workers and 17.6 % (n = 3 of 17) of veterinarians (*p* = 0.13). Tick-bites were mainly related to animal activities for farmers (50 %; *n* = 25), outdoor activities for rangers (73.5 %; n = 25), hobbies for veterinarians (56.3 %; *n* = 9) and pet activities for slaughterhouse workers (83.3 %; *n* = 5). The main tick-removal method reported by participants was “fingers” for farmers (62.3 %, *n* = 33) and slaughterhouse workers (75.0 %, *n* = 6) and “tick-removal tool” for rangers (62.5 %, n = 25). Of those bitten at least once in life, 1.8 % (n = 2) reported flu-like symptoms and 10.7 % (*n* = 12) erythema. Among the 25.9 % (*n* = 52) reporting involvement in hunting activities, 71.2 % (*n* = 37) reported eviscerating prey, with 54.1 % (*n* = 20) not taking necessary safety measures. Similarly, 85.6 % (*n* = 172) of participants reported to neglect taking precautions against tick-bites when outdoors (Table 2).

**Fig. 1:**
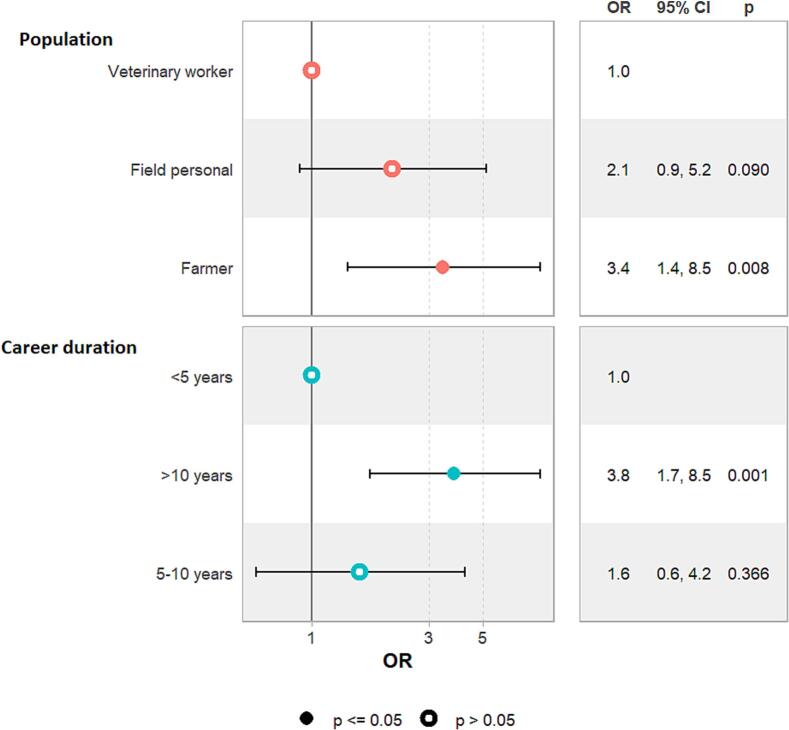
Forest plot showing association between occupational-related variables and tick-bite rates in high-risk populations (farmers, rangers and veterinarians).

**Fig. 2:**
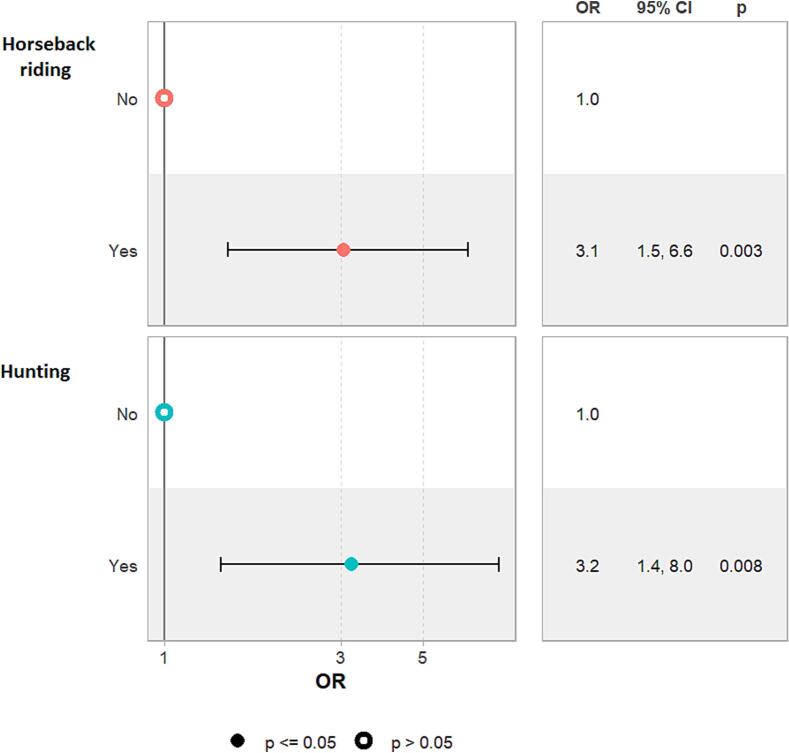
Forest plot showing association between non-occupational-related variables and tick-bite in high-risk populations (slaughterhouse workers, farmers, rangers and veterinarians).

The results of the multivariate analyses on risk factors for tick bite rates are illustrated in Fig. 1, Fig. 2. Tick bites rates, in terms of OR, were independently associated with farmers (OR = 3.4 [1.4–8.5]; *p* = 0.008) and high-risk participants with over 10 years of career duration (OR = 3.8 [1.7–8.5]; *p* = 0.001). Additionally, individuals engaged in horseback riding (OR = 3.1 [1.5–6.6]; *p* = 0.003) and hunting (OR = 3.2 [1.4–8.0]; p = 0.008) also had a significantly increased likelihood of tick bite rates. However, other variables considered in the multivariate analysis, including age, fishing, hiking, evisceration after hunt, tick body verification and awareness of tick-borne diseases, were not significantly associated with tick bite rates and were therefore excluded from the final model.

### 3.2 CCHFV seroprevalence

Anti-CCHFV antibodies seropositivity was of 0.08 % (*n* = 2) [95 % CI: 0.06–0.09] in RS, which was statistically different from the seropositivity estimated in high-risk groups, (0.50 % (*n* = 1); [95 % CI: 0.43–0.56] (*p* < 0.01). The two RS showing CCHFV antibodies seropositivity corresponded to a 76-year-old man and a 77-year-old woman, both living in Haute-Corse (northern Corsica). Neutralizing antibodies were not detected in these two samples. Among the 201 high-risk individuals, one was positive for CCHFV total antibodies and also positive for neutralizing antibodies at a 40 titer: he was a 53-year-old male slaughterhouse worker, employed for the past 5 to 10 years with no history of foreign travel to known CCHFV endemic areas, and also living in Haute-Corse. During his work, he used personal protective equipment (PPE) like boots and overall but not mask, gloves and protective glasses. He lacked prior knowledge of humans and animals TBDs and declared no outdoor activities such as hunting, hiking, horseback riding, or fishing. He reported not conducting routine body inspections, but approximately five previous tick bites, all of which were associated with domestic animal contact, specifically pets. None of these tick bites occurred within the 12 months preceding his fingertip blood sampling. He had previously experienced symptoms following a tick bite, including fever ranging from 37.5 to 39 °C but did not seek medical care.

## 4 Discussion

Here, we report evidence of exposure of the human population in Corsica to CCHFV. These results enhance the understanding of the epidemiological situation in Corsica following the recent detection of CCHFV RNA in ticks collected from cattle in 2023 [9], the evidence of exposure to CCHFV in ruminants [7] and the presence of *Hyalomma marginatum* in Corsica since 1950, where it is now well-established [8]. Altogether, these results are consistent with the emergence of CCHFV and with a risk of human exposure to CCHFV in Corsica.

Observed seroprevalence rate in high-risk populations is lower than that observed in Turkey, the most affected country in Europe, where rates can reach 12.8 % in endemic regions [15]. However, the rate observed in Corsican high-risk populations is consistent with those reported in Western European regions [4]. Similarly, the general population shows low seroprevalence, as evidenced by studies conducted in Spain, where rates range from 0 % to 1.6 % [[12], [16], [17]], even in areas with higher deer seroprevalences [16]. This further supports the notion of an epidemiological scenario characterized by high enzootic circulation in animal hosts and low seropositivity in humans.

Exposure rates to CCHFV in the human population do not necessarily reflect the risk of emergence of autochthonous cases, as shown by the situations in Greece and Spain. In Greece, the CCHF epidemiology is unique, as previous seroprevalence studies in the human population showed rates over 5 % without reports of clinical cases, except one fatal case reported in 2008 [18]. However, Spain reports a contrasting pattern, where low seroprevalence rates were found, despite over 10 autochthonous cases being reported [12]. The reasons for these differences are poorly understood and may be related to differences in pathogenicity or disease severity, which could be associated with the genetic heterogeneity of CCHFV, particularly among different lineages.

Evidence of human CCHFV seropositivity in the absence of clinical cases is not uncommon, even in regions where health professionals are familiar with CCHF diagnosis [19]. In Bulgaria, where the disease has been well-known for over 60 years, significant human seroprevalence rates have been observed even in regions where no human CCHF cases have been reported, assuming that many CCHF patients are asymptomatic or develop nonspecific symptoms. It is generally considered that CCHF should be viewed as a relatively rare event following CCHFV infection, with 80–90 % of cases either going unnoticed or misdiagnosed as “summer flu” [20].

Other examples with the absence or low reporting of clinical cases despite the presence of antibodies in human populations have been reported in Europe and the Maghreb. In Portugal, a seroprevalence study conducted before the 2000s revealed CCHFV seroprevalence rates of 1.1 % in the general human population [13]. In Hungary, where *Hyalomma* ticks have been present since 2012, antibodies were detected in 0.37 % of the general population from 2014 to 2020 [21]. In Tunisia, a seroprevalence of 5.2 % was observed among slaughterhouse workers in 2014 [22].

Taking into account the various epidemiological elements outlined above, the low seroprevalence rates reported in the Corsican population are not sufficient to assess the level of risk for the emergence of autochthonous cases (none has been reported to date), but nevertheless provide evidence of the risk of exposure.

There are several possible explanations for the lack of reported clinical cases despite seropositivity, including the circulation of a less-virulent strain leading to subclinical infections, as observed with strains circulating in Turkey and Greece [22]. Another potential explanation is the inadequate investigation of potential human cases, including limited awareness of the clinical manifestations of the disease and a lack of testing for CCHF in the absence of severe symptoms, which may lead to the underdiagnosis of milder cases. A similar situation was observed in Spain, where the first diagnosed case was reported in 2016; however, the virus had been circulating since 2010, and retrospective identification of a case from 2013 suggests that milder cases were likely present earlier [5]. These findings underscore the dynamic nature of CCHFV epidemiology and the importance of ongoing surveillance to monitor potential disease manifestation.

Finally, the presence of the Africa 1 genotype in Corsica [9] which differs from the Africa 3 genotype detected in southwestern France [23], raises questions about the pathogenicity attributed to CCHFV genotypes. The role of viral determinants in disease severity is unknown, and it is unclear how CCHFV genetic diversity contributes to the broad global range in the reported CFR of 2–80 % [24]. Heterogeneity, along with other factors such as availability of advanced medical care and host factors, may partially account for this variation. It is not impossible that different genotypes may exhibit different pathogenicity as previously observed with the AP92 strain in Greece, which is now reclassified as Aigai virus [25]. Analysis of CCHF severity in Russia according to the virus variant showed that risk of severe clinical manifestation, hemorrhagic syndrome and fatal outcome is increased for the CCHFV Europe-1 lineage compared with the Europe-3 and Africa-3 lineages [26]. Non-human primate models have demonstrated variable outcomes in CCHFV-infected macaques, influenced by the genetics of cynomolgus macaques, but also by differences in virus strains used and institutional variation in euthanasia criteria [3].

In our study, three samples showed seropositivity for antibodies by ELISA, one of which belong to a slaughterhouse worker who also tested positive for neutralizing antibodies. Previous studies have shown that neutralizing antibodies decline approximately five months after disease onset, exhibiting lower titers and reduced stability in the blood of survivors compared to CCHFV IgG, which can remain detectable for at least five to nine years post-recovery [27]. This enhances the likelihood of detection in samples, highlighting the epidemiological value of IgG-based surveillance. The slaughterhouse worker who tested positive for anti-CCHFV antibodies exhibited multiple risk factors, including rare use of PPE and history of tick bites. Abattoir workers face a fourfold higher risk of seropositivity compared to the general population due to the exposure to secretions and involvement in slaughtering [4]. Previous studies indicate that improper use of PPE increases the risk of infection [28], which is coherent with the case report mentioned above.

Globally, our study provided insights into the prevalence of tick bites among various occupational groups and the associated risk factors. The proportion of high-risk persons who have been bitten by a tick in their lifetime is higher than that reported by the Health Barometer 2019 survey, conducted in France [29]. This result strengthens the correlation between knowledge and protection. In our study, recreational activities such as hunting and horseback riding increased the likelihood of tick bite rates compared to those who did not engage in these activities. This observation aligns with previous research indicating that the more time spent outdoors the greater the risk of tick bites, with risks reported to increase by a factor of 1.1–1.2 for every hour spent outside [30]. Additionally, in Corsica, an area where CCHFV circulation is known to occur, it is crucial to highlight that, although self-reported surveys tend to underestimate the true incidence of tick bites, the exposure rate remains notably high. This elevated rate of exposure suggests a substantial risk for the population in terms of tick bites and the potential transmission of tick-borne viruses, including CCHFV.

We should acknowledge several limitations. First, one of the two included populations consisted of RS from patients undergoing medically prescribed blood tests (e.g., chronic diseases), hence the findings may not be representative of the general population. Second, convenience sampling is a potential source of bias and limits the representativeness of high-risk populations. Third, the small sample sizes may limit interpretation of results. Fourth, seroprevalence is a dynamic parameter, as some participants may lose antibodies over time and may appear seronegative despite a past CCHFV infection. Therefore, our results may underestimate the true circulation of CCHFV in Corsica. Fifth, our study shares the typical limitations of surveys based on self-reporting, including social desirability and recall bias. Consequently, with the use of closed questions, participants' declarations regarding compliance with preventive measures against tick bites or exposure to animal body fluids could have been overestimated. Key strengths of this real-world study include the use of an ELISA assay with excellent performance (sensitivity of 96.8–99.8 % (95 % CI) and specificity of 99.8–100 % (95 % CI)) [31], as well as the combination of both ELISA and seroneutralization assays to estimate the overall proportion of CCHFV infection. Another strength was the availability of sociodemographic, clinical and epidemiological data in high-risk groups.

## 5 Conclusion

Our study provides new evidence of CCHFV circulation in Corsica, supported by the detection of human antibodies, indicating its geographical expansion into this region. It also represents a crucial database for further research on the virus, essential for assessing whether the risk of exposure increases, decreases, or remains stable across different populations in the region. This highlights the necessity of conducting studies with larger high-risk human populations to evaluate CCHFV exposure risk in Corsica. These findings emphasize the need for ongoing surveillance, public health education, and preventive measures addressing both occupational and recreational factors in tick-borne disease prevention strategies. Livestock attendants and slaughterhouse workers should be informed of the potential risk of CCHFV infection and advised to adhere to strict hygiene practices when handling tick-infested animals. To adequately address the potential risk of viral hemorrhagic forms, health facilities must be equipped to diagnose and identify the virus, especially during active tick vector periods. All these points will be investigated in Southern France by a new consortium studying the factors contributing to CCHFV emergence in the human population. Enhancing preventive actions through citizen science and socio-ecological approaches will be one of the main objectives of the Assessing the Risk of Crimean-Congo Hemorrhagic fever Emergence in Southern France **“**ARCHE” project (PREZODE fundings).

## Additional information

PK is a PhD student at the Unité des Virus Emergents, University of Corsica Pascal Paoli and Aix-Marseille University, France. Her primary research interests focus on tickborne viruses via a One Health approach.

## CRediT authorship contribution statement

**Paloma Kiwan:** Writing – original draft, Methodology, Investigation, Formal analysis, Data curation, Conceptualization. **Morena Gasparine:** Data curation. **Dorine Decarreaux:** Data curation. **Lisandru Capai:** Data curation. **Shirley Masse:** Data curation. **Miša Korva:** Methodology. **Tatjana Avšič-Županc:** Methodology. **Jean Canarelli:** Resources. **Marie-Helene Simeoni:** Resources. **Xavier de Lamballerie:** Writing – review & editing, Validation. **Remi Charrel:** Writing – review & editing, Validation, Supervision. **Alessandra Falchi:** Writing – review & editing, Writing – original draft, Validation, Supervision, Project administration, Funding acquisition, Conceptualization.

## Funding

This work was supported in part by the ISIDORe project through the ISID_602b service (funding from the European Union's Horizon Europe Research & Innovation Programme, Grant Agreement no. 101046133).

## Declaration of competing interest

The authors declare that they have no known competing financial interests or personal relationships that could have appeared to influence the work reported in this paper.

## Data Availability

No data was used for the research described in the article.

## Appendix A Supplementary data

Supplementary material Socio-demographic characteristics of the participants from the general population. Image 1
