# Supplementary material for: Serological evaluation of Crimean-Congo Hemorrhagic fever in humans with High-Risk professional exposure and in residual sera collected in 2022-2023 across Corsica (France)
Source: One Health. 2025 Mar 25;20:101020. doi: 10.1016/j.onehlt.2025.101020 (PMC11995033; doi:10.1016/j.onehlt.2025.101020)
Supplement: Supplementary file 1 — Supplementary material Socio-demographic characteristics of the participants from the general population. [file mmc1.docx]

| **Additional file** |  |
| --- | --- |
| Socio-demographic characteristics of the participants from the general population. | |
| **Characteristic** | **N = 2,514***^1^* |
| **Age** | 52 [15 - 104] |
| **Age group** |  |
| 15-39 | 714 (28.4 %) |
| 40-49 | 416 (16.5 %) |
| 50-69 | 919 (36.6 %) |
| 70-89 | 410 (16.3 %) |
| >90 | 55 (2.2 %) |
| **Sex** |  |
| Women | 1,530 (60.9 %) |
| **Blood sample location** |  |
| Haute-Corse | 1,453 (57.8 %) |
| Corse du Sud | 1,061 (42.2 %) |
| *^1^* Median [Range]; n (%) | |
